# The Influence of Corticosteroid Injections on Postoperative Outcomes of Carpal Tunnel Release: A Systematic Review

**DOI:** 10.1055/s-0043-1769739

**Published:** 2023-08-02

**Authors:** Ali Kumaş, Milly van de Warenburg, Tinatin Natroshvili, Marius Kemler, Mahyar Foumani

**Affiliations:** 1 Department of Plastic, Reconstructive and Hand Surgery, Martini Hospital, Groningen, The Netherlands

**Keywords:** carpal tunnel, corticosteroid injection, outcome

## Abstract

**Background** Carpal tunnel syndrome can be treated with corticosteroid injections (CIs) and surgery. In this systematic review, the influence of previous CI on different postoperative outcomes after carpal tunnel release is evaluated.

**Methods** A systematic literature search using several databases was performed to include studies that examined patients diagnosed with carpal tunnel syndrome who received preoperative or intraoperative CIs.

**Results** Of 2,459 articles, 9 were eligible for inclusion. Four papers reported outcomes of preoperative and four outcomes of intraoperative CIs. One study evaluated patients who received both intraoperative and preoperative corticosteroids.

**Conclusion** Intraoperative CIs are associated with reduced postoperative pain after carpal tunnel release and support earlier recovery of the hand function that can be objectified in a faster median nerve conduction speed recovery and lower Boston Carpal Tunnel Questionnaire (BCTQ) scores. Using preoperative CIs did not lead to enhanced recovery after carpal tunnel release, and both preoperative and intraoperative CIs might be predisposing factors for infections.

## Introduction

Carpal tunnel syndrome (CTS) is a commonly occurring nerve entrapment disorder with a reported incidence rate of up to 2.3 cases per 100 person-years. 1 Symptoms mostly consist of tingling, numbness, or burning sensation following the median nerve path distally from the carpal tunnel. Of all conservative treatments, splinting and corticosteroid injection (CI) are the most notable ones. For some patients, the injection of a corticosteroid provides enough relief to CTS symptoms that they no longer need any further treatment. However, when failure of response to CI and for that matter other conservative treatments occurs, patients will likely eventually undergo surgical carpal tunnel release (CTR). 1 2

Even though multiple studies investigated the influence of CIs on CTR, the clear influence of pre- and postoperative CIs remains unclear.

Therefore, a systematic review was conducted to summarize the benefits and disadvantages of pre- and intraoperative CIs in patients with CTR.

## Methods

The PRISMA guidelines were followed during all stages of this systematic review. 3

### Search Methods

In collaboration with a professional independent librarian, multiple databases, MEDLINE, Embase, Web of Science, and PubMed, were systematically searched to find eligible articles on CTR and CIs ( Appendix A ). References lists of included studies were hand searched for relevant studies. There were no geographic or language restrictions. The last update on the search results was performed in September 2022. No contact was made with authors for unpublished data or results. The review of the titles, abstracts, and full-text articles were done independently by two researchers.

### Eligibility Criteria

Studies were included if they contained at least 10 clinically diagnosed patients with atraumatic CTS who underwent open CTR and underwent a pre- or intraoperative CI.

Studies were excluded based upon the following criteria: not acquirable in full text, or no clear description of postoperative results.

Endnote X5 was used during the processing of the search results. 4 Duplicate articles were deleted.

For the first round of reviewing, articles were included based upon their titles and abstracts. A third individual researcher acted as the third observer, who was consulted when a consensus was out of reach. Following this, all articles were under full text review to be checked for inclusion and exclusion criteria by both researchers.

### Quality Assessment

Depending on study type, quality assessment and risk-of-bias evaluation were done by two reviewers. A modified Cochrane risk-of-bias tool (RoB 2) was used for the randomized trials. 5 After scoring the RoB 2, an overall risk-of-bias judgment of either low, some concerns, or high was made ( Table 1 ). Quality assessment of the cohort studies was done using the Newcastle-Ottawa Scale (NOS). 6 The finalized result of the NOS is a score ranging from 1 to 9 stars, with 7 to 9 stars being regarded as high quality, 4 to 6 moderate quality, and 1 to 3 low quality ( Table 2 ).

**Table 1:** Characteristics of included intraoperative corticosteroid injection studies and risk-of-bias assessment

Author	Title	Study type	Risk-of-bias assessment	Funding or conflict of interest
Padua et al 2003 8	Intrasurgical use of steroids on carpal tunnel syndrome: a randomized, prospective, double-blind controlled study	Prospective randomized controlled trial	Low	Nm
Stepić et al 2008 10	Effects of perineural steroid injections on median nerve conduction during the carpal tunnel release	Prospective randomized controlled trial	Some concerns	Nm
Naji 2011 9	Intraoperative steroid irrigation in carpal tunnel	Prospective randomized controlled trial	High	Nm
Mottaghi et al 2019 7	Carpal tunnel release surgery plus intraoperative CI versus carpal tunnel release surgery alone: a double blinded clinical trial	Prospective randomized controlled trial	Low	Nm

Abbreviation: Nm, not mentioned.

**Table 2:** Characteristics of included preoperative corticosteroid injection studies and quality assessment

Author	Title	Study type	Quality assessment	Funding or conflict of interest
Hanssen et al 1989 14	Deep postoperative wound infection after carpal tunnel release	Retrospective cohort	Poor	Nm
Edgell et al 2003 13	Predicting the outcome of carpal tunnel release	Retrospective cohort	Poor	Yes, funded by Jewish Hospital HealthCare Services, the University of Louisville, and the Christine M. Kleinert Institute for Hand and Microsurgery 13
Vahi et al 2013 11	Preoperative CIs are associated with worse long-term outcome of surgical carpal tunnel release	Retrospective cohort	Poor	None
Bland and Ashworth 2015 12	Does prior local CI prejudice the outcome of subsequent carpal tunnel decompression?	Retrospective cohort	Poor	None
Kirby et al 2021 15	Influence of corticosteroid injections on postoperative infections in carpal tunnel release	Retrospective cohort	Good	Nm

Abbreviation: Nm, not mentioned.

### Data Extraction

The data of the studies were independently extracted by two researchers. From the studies, the following data were extracted: patient population, demographics, number of injections for each patient, duration between injection and surgery, type and dosage of injection, number of infections, functional outcome report, nerve conduction measurements, Boston Carpal Tunnel Questionnaire (BCTQ) scores, and eventually other relevant outcomes ( Table 3 ).

**Table 3:** Summary of data of studies included

Author	Moment of injection	No. of patients injected ( *n* )	Mean age (y)	Male/female (%)	No. of injections ( *n* )	Duration between injection and surgery (d)	Injection	Dosage
Edgell et al 13	Preoperative	54 (57)	47.8	24 (42)/33 (58)	1: 29 2: 20 ≥3: 5	Nm	Betamethasone	3 mg
Vahi et al 11	Preoperative	127 (174)	60.0	22 (11)/149 (89)	1: 31 2: 34 3: 35 4: 7 5: 7 9: 2 10: 7	Nm	Nm	Nm
Bland and Ashworth 12	Preoperative	278 (942)	62.0	293 (31)/649 (69)	1: 180 2: 79 3: 16 4: 3	269 d	Triamcinolone	40 mg
Kirby et al 15	Preoperative	32 (139)	63.4	35 (25)/104 (75)	1: 135 Multiple: 4	105 d	Dexamethasone	3.6 mg
Padua et al 8	Intraoperative	10 (20)	54.3	2 (10)/18 (90)	1: 10	After surgical depression, before skin closure, irrigation of the nerve	Methylprednisolone	40 mg
Naji 9	Intraoperative	20 (40)	38.5	2 (5)/38 (95)	1: 20	One minute before closure of the wound, irrigation of the wound	Methylprednisolone acetate (Depo-Medrol)	40 mg
Mottaghi et al 7	Intraoperative	20 (42)	46.2	2 (5)/40 (95)	1: 20	Before suturing the skin in the flexor tendon sheath and inside the borders of transverse carpal ligament incision and around the median nerve	Dexamethasone	1 mg
Stepi *ć* et al 10	Intraoperative	20 (40)	51.6	13 (32.5)/27 (67.5)	1: 20	During surgery immediately after decompression, a perineural injection	Betamethasone	1 mL
Hanssen et al 14	Pre- and intraoperative	Preoperative 16 (119) Intraoperative 59 (119)	56.0	77 (65)/42 (35)	Partly mentioned: 1: 74 2: 1	Preoperative timing Nm and intraoperative	Nm	Nm

Abbreviation: Nm, not mentioned.

### Outcomes

The primary outcomes of this study are infection and nerve conduction rates for patients receiving either pre- or intraoperative CI and for control patients. Secondary outcomes are any patient-reported outcomes with well-described outcomes before and after treatment and other CTS-related complaints.

## Results

**Fig. 1:**
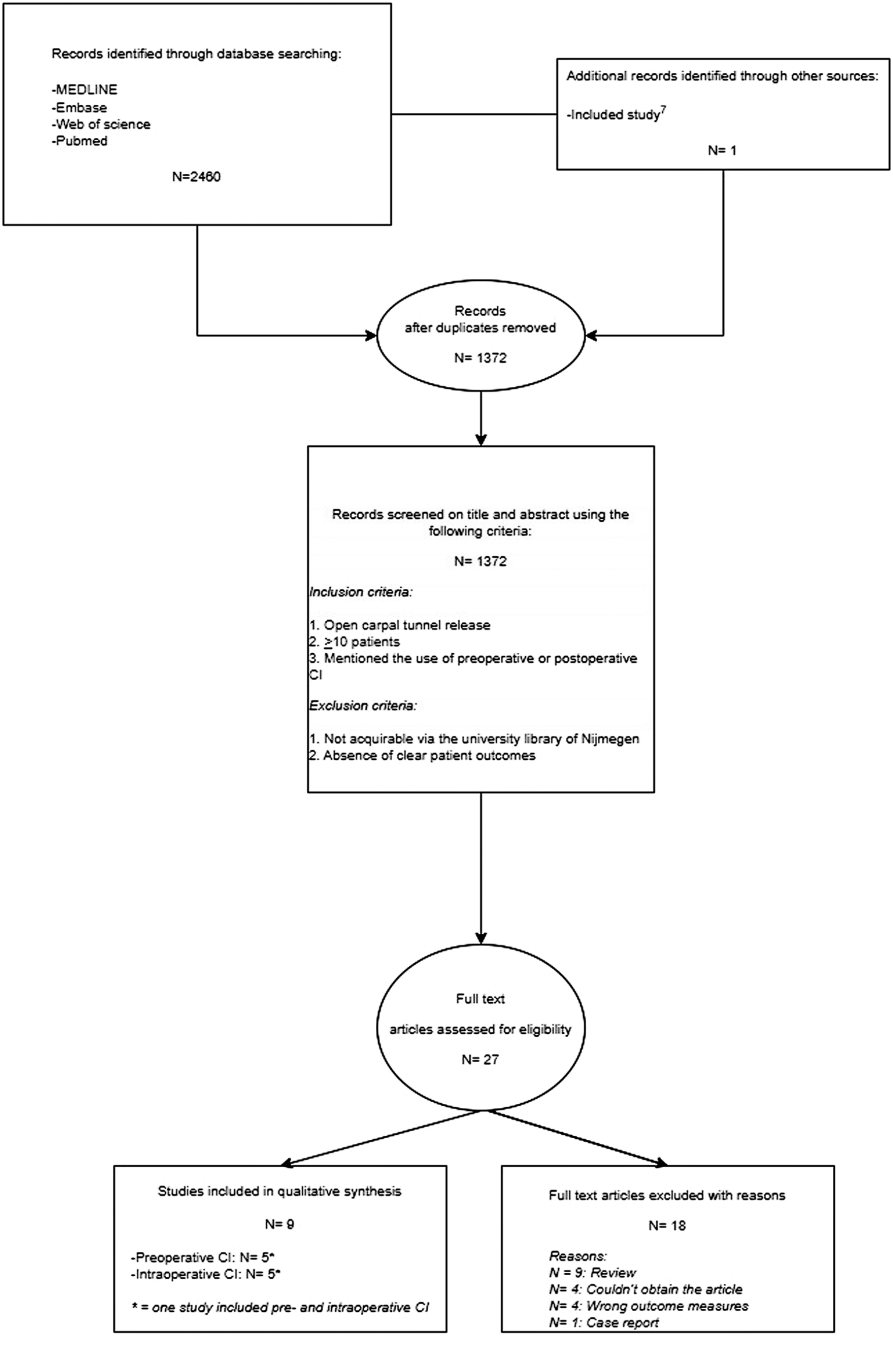
Flow chart of reviewing process.

The database search identified 2,470 papers, and 1 other paper was added through scanning the reference lists of included articles. 7 A total of 1,372 papers were left after removing duplicates and these papers were screened for eligibility on title and abstract. For the full text screening, 51 papers were assessed, leaving 9 relevant studies: 5 papers with intraoperative CIs and 5 papers with preoperative CIs. 7 8 9 10 11 12 13 14 15 One study used both intraoperative and preoperative CIs. 14 The reviewing process is shown in Fig. 1 .

Four of five studies using intraoperative CIs were randomized controlled trials, and one study was a retrospective cohort study. This study also included a preoperative CI cohort. 14 A summary of a risk of bias is given in Table 1 . All five studies using preoperative CIs were retrospective cohort studies. A summary of the quality assessment of these articles is given in Table 2 .

### Intraoperative Corticosteroid Injections

The five studies included in the intraoperative CI consisted of four randomized controlled trials and one retrospective trial, in which a total of 245 patients were included. Intraoperative CI was performed on 129 patients, and 116 patients received no injection. The CIs that were used and patient characteristics are shown in Table 3 .

#### Infections

Four of the included studies quantified the rate of wound infections after CTR. 7 8 9 14 In three of these studies, none of the 50 patients with an intraoperative CI developed a postoperative wound infection. 7 8 9 Hanssen et al reported 13 (22%) postoperative infections in the intraoperative CI group and 2 (4.5%) in the control group ( Table 4 ). 14 The intraoperative CI group had a significant ( *p* < 0.002) higher risk of having an infection. There was a difference between incidence of infections in male (0.87%) and female (0.25%) patients, which was also significant ( *p* < 0.02). In addition, a total of 19 patients received prophylactic antibiotics, which did not change the statistical analysis; the difference in postoperative wound infections between the intraoperative CI and control group was still significant.

**Table 4:** Infections after intraoperative corticosteroid use in Hanssen et al 14

Group	Patients	Number of infections (%)	Revisions ( *n* )	Clinical presentation intraoperative CI ( *n* )
Intraoperative injection group	59	13 (22)	Surgical debridement (14), debridement of infected tendon sheaths (6)	● Carpal canal pain (8) ● Small finger pain (3) ● Spontaneous drainage (2) ● Thumb (flexor) pain (2)
Control group	44	2 (4.5)		
Total	103	15 (14.6)		

Abbreviation: CI, corticosteroid injection.

#### Postoperative Pain

Only one of the included studies reported on postoperative pain after CTR. 9 Naji evaluated postoperative pain after CTR in 40 patients. In total, 20 patients received an intraoperative CI and none of them (0%) reported postoperative pain complaints. In the control group of 20 patients, 11 patients (55%) had persisting agonizing pain postoperatively at the ulnar side of the wrist (incision site), which was not responding to analgesic treatment. The other nine patients (45%) did not have any postoperative pain complaints. This difference was found to be significant ( *p* < 0.05).

#### Symptom Relief

**Fig. 2:**
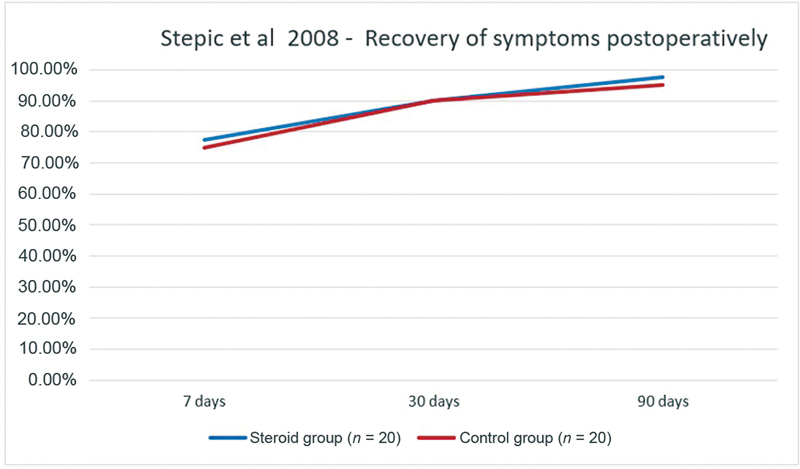
Postoperative recovery of symptoms in patients (%) after carpal tunnel release in Stepić et al.

One study described postoperative symptom relief of CTS. 10 Stepić et al registered the disappearance of symptoms in 40 patients after CTR, of which 20 patients received an intraoperative CI. After 3 months, only one patient (5%) receiving intraoperative CI still experienced symptoms, compared with two patients (10%) in the control group. Intraoperative CI did not lead to an increased proportion of patients without symptoms when compared with patients receiving no CI ( Fig. 2 ).

#### Median Nerve Conduction Speed

Two studies performed electrophysiological measurements in a total of 82 patients before and after CTR, comparing 40 patients receiving an intraoperative CI with 42 patients in a control group without CI. 7 10 The median (sensitive) nerve conduction speed and the distal motor and sensory latencies were measured. In 20 patients receiving intraoperative CI, a faster and bigger recovery in electrophysiological measurements was established, compared with the control group.

**Fig. 3:**
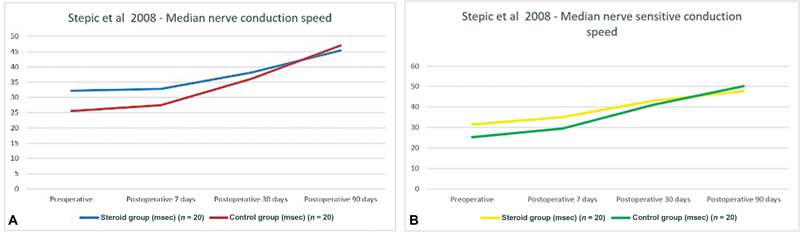
( **A, B** ) Median nerve (sensitive) conduction speed (ms) pre- and postoperatively in Stepić et al.

In Stepić et al, values of the median nerve conduction speed and sensitive conduction speed were measured both preoperatively and postoperatively after 7, 30, and 90 days. 10 Twenty patients receiving intraoperative CI were compared with 20 patients in the control group ( Fig. 3a, b ). The median nerve conduction speed measurements 7 days postoperatively were not significantly different between these groups. After 30 days, the median nerve conduction speed improved in both groups, but this improvement was significantly greater in the CI group ( *p* = 0.025). After 90 days, both groups showed improvement of the median nerve conduction speed in comparison to the preoperative situation, which was significantly greater in the CI group ( *p* = 0.043).

In Mottaghi et al, the median sensitive conduction speed after 30 days also improved significantly in the CI group compared with the control group ( *p* = 0.018). 7 The measurements after 90 days did not show any significant difference between the CI group and the control group.

**Fig. 4:**
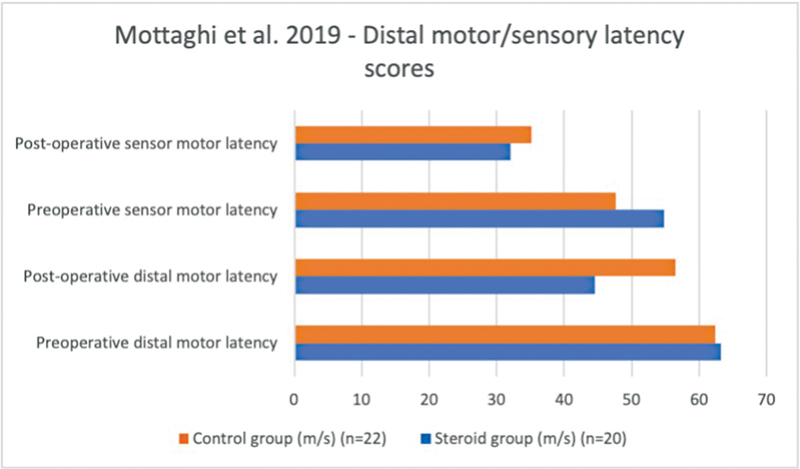
Motor and sensory latency values (m/s) in Mottaghi et al.

Furthermore, the nerve conduction speed in the distal motor and sensory nerves showed a greater improvement in the intraoperative CI group compared with the control group ( Fig. 4 ). 7 The distal motor latency improved by 30.0% in the steroid group and by 25.3% in the control group, compared with the preoperative situation. The distal sensor latency improved by 37.7% in the steroid group and by 26.3% in the control group. However, the data were not significantly different (preoperative *p* = 0.671, postoperative 7 days *p* = 0.404, postoperative 30 days *p* = 0.16, postoperative 90 days *p* = 0.977).

#### Boston Carpal Tunnel Questionnaire

In two studies, pre- and postoperative BCTQ scores were compared in a total of 62 patients: 30 intraoperative CI patients and 32 control patients. 7 8 The improvement in the BCTQ scores of the CI groups was shown in both studies. The mean BCTQ score improved by 63.3% in 30 patients with intraoperative CI and by 48.7% in 32 patients without CI.

**Fig. 5:**
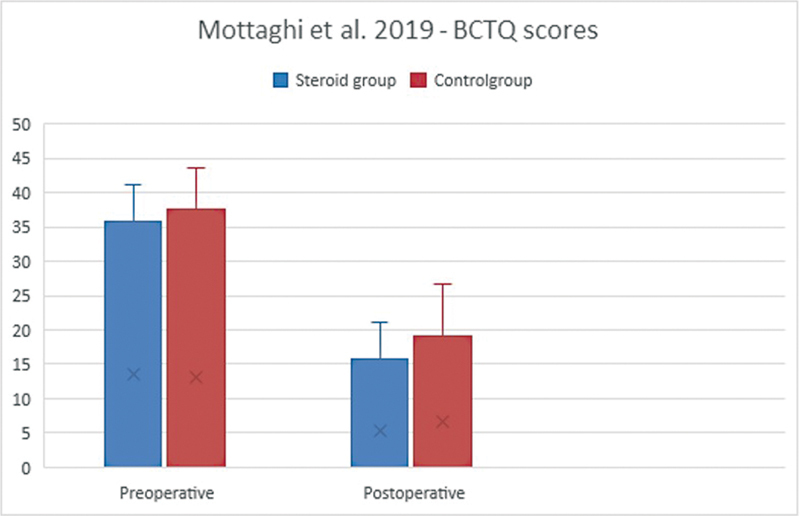
Preoperative and postoperative BCTQ scores in Mottaghi et al. BCTQ, Boston Carpal Tunnel Questionnaire.

The first study, Mottaghi et al, used the sum of the symptom and functional scales to determine a BCTQ index ( Fig. 5 ). 7 Both groups showed improvement of symptoms ( *p* < 0.001) after CTR, but the CI group showed greater improvement (the mean BTCQ improved with 55.8%) compared with the control group (mean BTCQ improvement of 49.2%). However, the postoperative values were not significantly different from one another ( *p* = 0.131).

**Fig. 6:**
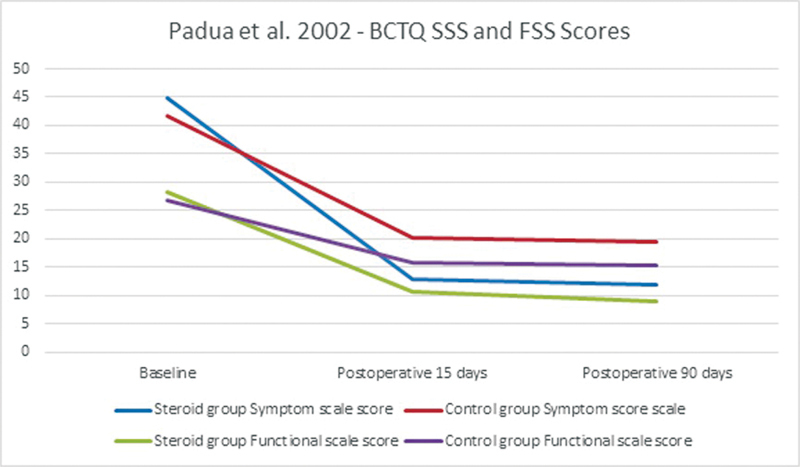
BCTQ symptom and functional scales in Padua et al. BCTQ, Boston Carpal Tunnel Questionnaire; FSS: Functional Status Scale; SSS, Symptom Severity Scale.

Padua et al also used the BCTQ index but showed the values in scales: the Symptom Severity Scale (SSS) and Functional Status Scale (FSS) of the BCTQ ( Fig. 6 ). 8 In 2 months, the SSS improved by 73.4% in the CI group and by 53.6% in the control group, compared with the preoperative scores. The FSS improved by 68.1% in the CI group and by 42.8% in the control group. The changes from baseline to 15 and 60 days showed a significant difference in the SSS in both groups ( *p* = 0.0035 and 0.005, respectively) compared with the preoperative scores. The postoperative FSS differences were not significant between the study groups.

### Preoperative Corticosteroid Injections

The five studies included in the preoperative CI group were all retrospective cohort studies, in which a total of 1,413 patients were included. 11 12 13 14 15 From these patients, 504 patients underwent a preoperative CI, and 909 patients did not receive a CI. Study characteristics are shown in Table 3 .

#### Infection

Two studies mentioned if any infection occurred. Hanssen et al reported two (12.5%) infections in the preoperative CI group and two (4.5%) in the control group ( Table 5 ). 14 There was no statistically significant correlation between preoperative CI and infection in this study. There was a difference between incidence of infections in male (0.87%) and female (0.25%) patients, which was significant ( *p* < 0.02). In total, 19 patients received prophylactic antibiotics, which did not change the statistical analysis; the difference was still not significant.

**Table 5:** Overview of results of intraoperative corticosteroid injections

Article	**Patients with infection (% of total patients)**		**Boston Carpal Tunnel Questionnaire**				**Patient-reported outcomes**	
**Symptom score**		**Functional score**	
**CI**	**Control**	**CI**	**Control**	**CI**	**Control**	**CI**	**Control**
Hanssen et al 14	2 (12.5%)	2 (4.5%)	–	–	–	–	–	–
Kirby et al 15	16 (41%)	16 (16%)	–	–	–	–	–	–
Bland and Ashworth 12	Nm	Nm	1.42	1.47	1.45	1.45	Success rate (in %):	
							84	85
							Failure rate (in %):								
							3.9	3.3
Vahi et al 11	Nm	Nm	–	–	–	–	NRS (in points):	
							88	84

Abbreviations: CI, corticosteroid injection; Nm, not mentioned; NRS, Numeric Rating Scale.

Kirby et al analyzed patients with postoperative infection retrospectively and compared these to control patients ( Table 5 ). 15 Patients in the infection group had a significantly shorter period between CI and surgery (77 ± 52 days) than in the control group (133 ± 89 days) ( *p* = 0.05). Thirty-six patients (92%) had a superficial postoperative infection, and three patients (8%) had a deep postoperative infection ( Table 6 ).

**Table 6:** Infections after preoperative corticosteroid use in Hanssen et al 14 and Kirby et al 15

Group	Patients without infection (%)	Number of infections (%)	Revisions in Hanssen et al 14 ( *n* )	Clinical presentation in Hanssen et al 14 ( *n* )
Preoperative injection	30 (19.2)	18 (41.9)	Surgical debridement (14), debridement of infected tendon sheaths (6)	● Carpal canal pain (10) ● Small finger pain (5) ● Spontaneous drainage (2) ● Thumb (flexor) pain (2)
Controls	126 (80.8)	25 (58.1)		
Total	156 (100)	43 (100)		

**Fig. 7:**
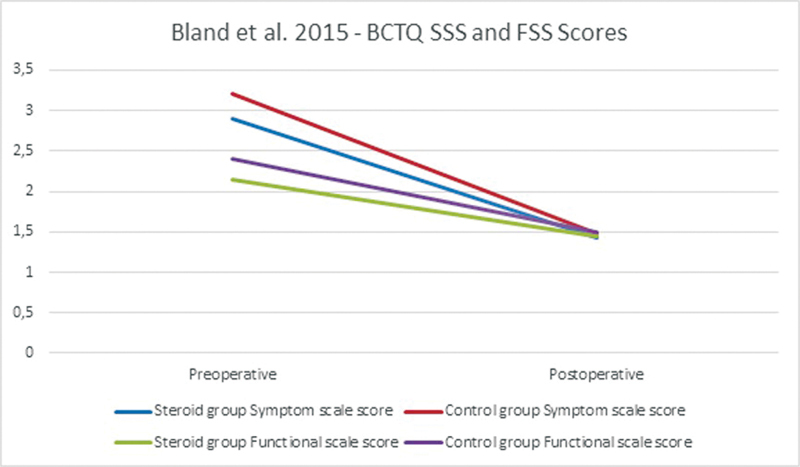
BCTQ SSS and FSS scores preoperatively and postoperatively in Bland and Ashworth. BCTQ, Boston Carpal Tunnel Questionnaire; SSS, Symptom Severity Scale.

Only one study reported on BCTQ scores in patients receiving preoperative CIs. Bland and Ashworth used the BCTQ to measure the symptom severity and functional status in 942 patients: 278 patients underwent a preoperative CI and 664 patients did not ( Fig. 7 ). 12 The postoperative SSS and FSS scores showed more improvement in the control group when compared with the preoperative corticosteroid group but the improvements were not significant (SSS *p* = 0.37 and FSS *p* = 0.40).

### Patient-Reported Outcomes

Two studies used patient-reported outcomes of 1,170 patients to evaluate the success of preoperative CI. 11 12 A total of 182 patients (39.7%) receiving a preoperative CI were free of symptoms postoperatively, compared with 268 patients (37.7%) in the control group. Also, 277 patients (60.3%) receiving a preoperative CI still experienced symptoms such as pain and paraesthesia postoperatively, compared with 443 patients (62.3%) in the control group. Bland and Ashworth found no significant difference in patient-reported outcomes after CTR in the injection group compared with the control group ( *p* = 0.82). 12

Vahi et al found that patients who received a preoperative CI had a higher occurrence of complaints (pain [ *p* = 0.04], paraesthesia [ *p* = 0.007], and nocturnal awakenings [ *p* = 0.003]) when compared with the control group. 11 Within the injection group, there was an association between patients receiving more injections and the reporting of the specific complaints. However, this difference was not significant ( *p* = 0.09). Other studies did not mention if there were any pain complaints postoperatively.

In Edgell et al, patients were considered cured if they did not have any complaints after 6 months postoperatively. 13 In total, 13 patients (87%) with some relief after CI underwent surgery, while 21 patients (54%) without relief after CI underwent surgery.

In Vahi et al, the patients were also asked to fill in the Numeric Rating Scale (NRS) from 0 to 100: 100 points meaning total regression of symptoms and 0 meaning no effect. 11 In total, 120 patients had regression of symptoms, meaning they scored 90 or more points in the NRS. In the control group, 33 patients (70%) had a score of 90 or more points, while in the steroid group 86 patients (68%) scored 90 points or more. The pain was still present in 14 patients (30%) in the control group and in 41 patients (32%) in the steroid group. The difference in NRS scores between patients receiving preoperative injections and the control group was not significant.

### Nerve Conduction Studies

Edgell et al measured median sensory values of 31 patients with preoperative CI, in which 24 (77%) patients had diminished values, 16 (67%) patients were symptom-free after surgery, and 7 (23%) patients had a normal median sensory value; 3 (43%) of these patients showed complete relieve by CTR. 13 The number of patients who were symptom-free after CTR did not differ significantly between the group with retarded and normal median sensory values preoperatively ( *p* = 0.255). The median motor measurement was performed on 37 patients: 24 (65%) patients had a diminished value, of whom 15 (63%) recovered after CTR, and 13 (35%) patients had a normal value, of whom 6 (46%) recovered after CTR ( *p* = 0.338). The number of patients who were symptom-free after CTR was not significantly different between the groups with retarded or normal median motor measurements preoperatively ( Table 7 ).

**Table 7:** Overview of results of preoperative corticosteroid injections

Article	**Infection**		**Postoperative pain**		**Symptom relief (in %)**		**Electrodiagnostic measurement**		**Boston Carpal Tunnel Questionnaire**	
**CI**	**Control**	**CI**	**Control**	**CI**	**Control**	**CI**	**Control**	**CI**	**Control**
Hanssen et al 14	13 (22%)	2 (4.5%)	–	–	–	–	–	–	–	–
Naji 9	0 patients with infection in both groups		0 (100%)	11 (55%)	–	–	–	–	–	–
Stepić et al 10	Nm	Nm	Nm	Nm	7 d postoperative:		Sensitive conduction speed preoperative:		–	–
					77.5	75	31.493	25.287		
					30 d postoperative:		Sensitive conduction speed 90 d postoperative:			
					90	90	47.673	50.147			
					90 d postoperative:		Conduction speed preoperative:			
					97.5	95	32.2	25.487		
							Conduction speed 90 d postoperative:			
							45.347	47.027		
Mottaghi et al 7	0	Nm					Predistal sensory latency:		Questionnaire sum preoperative:	
							54.83	47.6	35.83	37.7
							Postdistal sensory latency:		Questionnaire sum postoperative:	
							34.11	35.1	15.83	19.15
							Predistal motor latency:			
							63.22	62.35		
							Postdistal motor latency:			
							44.56	46.55		
Padua et al 8	0 patients with infection in both groups		–	–	–	–	–	–	Symptoms score at baseline:	
									44.8	41.8
									Symptoms score after 60 d:	
									11.8	19.4
									Functional score at baseline:	
									28.2	26.9
									Functional score after 60 d:	
									9	15.4

Abbreviations: CI, corticosteroid injection; Nm, not mentioned.

## Discussion

In this systematic review, nine studies were evaluated for the outcome of CTR after either preoperative or intraoperative CIs. 7 8 9 10 11 12 13 14 15 A meta-analysis could not be performed on any of the outcome measures due to the lack of data and heterogeneity of the data in the included studies.

Administering intraoperative CI might be beneficial for reduced postoperative pain after CTR and lead to earlier recovery of the hand function. 9 10 However, no benefit is seen in using intraoperative CI for the final outcomes in terms of symptoms, function, and electrophysiology. 7 8 10

Hanssen et al found that an intraoperative CI increases the risk of having a postoperative infection compared with the control group. 14 This study, however, associates the infection rates with extended operative techniques, such as flexor tenosynovectomy and the insertion of a surgical drain, and not with the steroid usage. Other studies using intraoperative CIs did not find any infection in their cohorts after CTR, leading to believe the usage of this injection does not predispose patients to postoperative infections. 7 8 9 10 In Mottaghi et al, all patients received postoperative prophylactic cefazolin 500 every 6 hours for 3 days, which can cause unnecessary antibiotic resistance due to the fact that intraoperative CI does not significantly induce infections. 7

Kirby et al showed that the usage of preoperative CI increases the risk of having a postoperative infection significantly. 15 However, the number of infections seems to be underrated, due to limited reporting or diagnosis of postoperative wound infections in different clinics. They recommend informing patients about the risk of infection after CTR when CIs are given in the prior 2 to 3 months. 15 Other studies using preoperative CIs did not mention infection rates. 11 12 13

There is no evidence for improved recovery after CTR when using preoperative CIs. Specifically, Bland and Ashworth found no difference in postoperative complaints after CTR between patients receiving a preoperative CI and the control group. 12 A possible advantage of preoperative CI could be a holdup for a CTR. Disadvantages of this study were the small sample size, a big loss in follow-up, and heterogeneity between the study groups due to the direct offer to undergo CTR to patients with serious complaints. Vahi et al found that preoperative CIs lead to more postoperative complaints, such as pain, paresthesia, and numbness. 11 However, the outcome of BCTQ scores, symptom relief, and NRS scores was not significantly better in the group of patients with preoperative CI, compared with the control group. 11 12 13 In conclusion, there is no evidence for improved recovery after CTR when using preoperative CIs. Additionally, the adverse effects of CIs such as skin depigmentation and subcutaneous atrophy should not be forgotten. 16

A limitation of this systematic review is the heterogeneity of the included studies, especially in the preoperative CI studies. The time between injection and surgery are different in all the studies, such as the number of CI per patient. Furthermore, the quality of four of the included studies addressing preoperative CIs is poor. A prospective randomized trial with large cohort of patients and a longer follow-up in the future could increase our knowledge of CI usage in patients after CTR.

**Appendix A:** Search strategy

No.	Search	MEDLINE hits
#1	(exp Carpal Tunnel Syndrome/OR carpal tunnel.ti,ab,kf. OR CTR.ti,ab,kf. OR CTS.ti,ab,kf. OR (Median nerve*.ti,ab,kf. AND (compress*.ti,ab,kf. OR entrap*.ti,ab,kf. OR neuropath*.ti,ab,kf.)) OR median neuropath*.ti,ab,kf. OR median neurit*.ti,ab,kf. OR (carpal.ti,ab,kf. AND (nerve entrapment.ti,ab,kf. or nerve compression.ti,ab,kf. or entrapment neuropath*.ti,ab,kf.)))	23,810
#2	(exp Steroids/OR exp Glucocorticoids/OR steroid*.ti,ab,kf. OR corticosteroid*.ti,ab,kw. OR glucocorticoid*.ti,ab,kf. OR exp Injections/OR inject*.ti,ab,kf.)	1,930,958
#3	(exp Postoperative Complications/OR exp Treatment Outcome/OR outcome*.ti,ab,kf. OR postoperat*.ti,ab,kf. OR post-operat*.ti,ab,kf. OR postsurg*.ti,ab,kf. OR post-surg*.ti,ab,kf. OR exp infections/OR infect*.ti,ab,kf.)	6,155,074
#4	#1 AND #2 AND #3	561
**Embase search**		
**No.**	**Search**	**Embase hits**
#1	(exp Carpal Tunnel Syndrome/OR carpal tunnel.ti,ab,kw. OR CTR.ti,ab,kw. OR CTS.ti,ab,kw. OR (Median nerve*.ti,ab,kw. AND (compress*.ti,ab,kw. OR entrap*.ti,ab,kw. OR neuropath*.ti,ab,kw.)) OR median neuropath*.ti,ab,kw. OR median neurit*.ti,ab,kw. OR (carpal.ti,ab,kw. AND (nerve entrapment.ti,ab,kw. or nerve compression.ti,ab,kw. or entrapment neuropath*.ti,ab,kw.)))	36,999
#2	(exp Steroids/OR exp Glucocorticoids/OR steroid*.ti,ab,kw. OR corticosteroid*.ti,ab,kw. OR glucocorticoid*.ti,ab,kw. OR exp Injections/OR inject*.ti,ab,kw.)	2,575,275
#3	(exp Postoperative Complications/OR exp Treatment Outcome/OR outcome*.ti,ab,kw. OR postoperat*.ti,ab,kw. OR post-operat*.ti,ab,kw. OR postsurg*.ti,ab,kw. OR post-surg*.ti,ab,kw.	4,400,647
#4	#1 AND #2 AND #3	1,041
**Web of Science search**		
**No.**	**Search**	**Web of Science hits**
#1	TS = (carpal tunnel* OR CTR OR CTS OR (Median nerve* AND (compress*OR entrap* OR neuropath*)) OR median neuropath* OR median neurit* OR (carpal AND (nerve entrapment OR nerve compression OR entrapment neuropath*)))	36,515
#2	TS = (Steroid* OR corticosteroid* OR glucocorticoid* OR inject*)	1,348,295
#3	TS = (*outcome* OR postoperat* OR post-operat* OR postsurg* OR post-surg* OR infect*)	4,472,112
#4	#1 AND #2 AND #3	564
**PubMed search**		
**No.**	**Search**	**PubMed hits**
#1	Search: ((“Carpal Tunnel Syndrome”[Mesh]) OR (“Carpal Tunnel Syndrome/surgery”[Mesh])) OR (“Carpal Tunnel Syndrome/drug therapy”[MAJR])	8,713
#2	Search: ((((((carpal tunnel[tiab]) OR (Carpal tunnel syndrome[tiab])) OR (CTS[tiab])) OR (Median nerve compression[tiab])) OR (Carpal tunnel release[tiab])) OR (open carpal tunnel release[tiab])) OR (carpal tunnel surgery[tiab])	17,211
#3	#1 or #2	18,554
#4	Search: ((“Steroids/administration and dosage”[MeSH]) OR (“Injections”[Mesh])) OR (“Injections, Intra-Articular”[MeSH])	378,459
#5	Search: (Steroid injection[tiab]) OR (Corticosteroids[tiab])	72,977
#6	#4 or #5	442,419
#7	Search: (“Treatment Outcome”[MeSH]) OR (“Postoperative Complications”[Mesh])	1,534,116
#8	Search: (infection[tiab]) OR (post-operative outcomes[tiab])	1,148,374
#9	#7 or #8	2,572,503
#10	#3 and #6 and #9	162
